# Editorial: Unifying the Global Surgical Community

**DOI:** 10.1055/s-0044-1789192

**Published:** 2024-08-14

**Authors:** Dale Dangleben

**Affiliations:** 1 Surgical Critical Care, Penn State Health, 348 North 24 Street, Camp Hill, PA

**Dale Dangleben:**
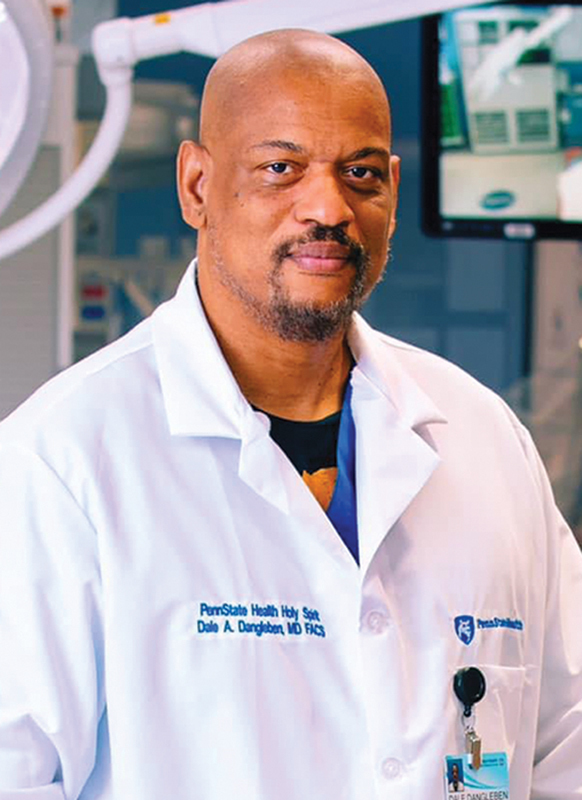


I am Dr. Dale A. Dangleben the Chief of Surgery and Trauma Medical Director at Penn State Health Holy Spirit Medical Center in Pennsylvania. In addition, I am an author and editor who is deeply committed to medical education and health care advocacy from a global perspective.

In the ever-evolving landscape of health care, the need for unity, inclusivity, and a global perspective in the field of surgery has never been more pressing. As we inaugurate this new chapter for our esteemed journal, I am both honored and excited to share our renewed vision and commitment to fostering a comprehensive, inclusive, and collaborative platform that brings together all members of the surgical community from across the globe.

Our journal's new focus is rooted in the belief that surgery is a universal language, transcending borders and cultures. With this global outlook, we aim to provide a platform that not only highlights the latest advancements in surgical specialties from around the world but also facilitates the exchange of knowledge and experiences across diverse health care systems. By embracing a global perspective, we can learn from each other, adopt best practices, and innovate collectively to improve surgical outcomes for patients everywhere.

Historically, surgical journals have primarily catered to surgeons, often overlooking the invaluable contributions of other health care professionals. We believe that true progress in health care comes from collaboration and inclusivity. Therefore, our journal will prominently feature contributions from not only surgeons but also from nurses, medical students, and other allied health professionals. Each member of the health care team plays a critical role in patient care, and their voices and insights are essential to advancing our understanding and practice of surgery.

Our mission is to unify the global surgical community in the pursuit of education, research, and improved patient outcomes. By providing a platform for diverse perspectives, we can foster a richer, more comprehensive understanding of surgical care. Our journal will feature relevant research, educational articles, case studies, and reviews that cater to the needs of a broad audience, from seasoned surgeons to those just beginning their medical journey.

As we embark on this journey together, we remain committed to maintaining the highest standards of excellence and innovation. Our editorial board comprises leading experts from the United States, Europe, Africa, the Caribbean, and beyond, ensuring a diverse and well-rounded representation of the global surgical community. We are dedicated to promoting rigorous, peer-reviewed research and providing a platform that contributes to the evolution of medical practices in light of changing technology.

We invite you to join us in this endeavor. Whether you are a surgeon, nurse, medical student, or allied health professional, your contributions are vital to the success of our journal and the advancement of surgical care worldwide. Together, we can create a dynamic and inclusive forum that will truly bring out the mission of this effort.

I extend my heartfelt gratitude to our readers, contributors, editors, and editorial board members for their unwavering support and dedication. As we turn the page to this new chapter, let us embrace the spirit of unity and collaboration in the name of better medical outcomes for all or patients. In spite of geography, let us all continue to learn together.

Thank you for being a part of this exciting journey.

Dale Dangleben, MD, FACS

